# Integrating germline and somatic genomic analysis to probe etiological mechanism in malignant glioma

**DOI:** 10.18632/oncotarget.26897

**Published:** 2019-05-03

**Authors:** Quinn Ostrom, Melissa L. Bondy, Jason T. Huse

**Affiliations:** Melissa L. Bondy: Department of Medicine, Section of Epidemiology and Population Sciences, Dan L. Duncan Comprehensive Cancer Center, Baylor College of Medicine, Houston, Texas, USA

**Keywords:** glioma, epidemiology, family history, molecular classification, genetic susceptibility

Genomic characterization of sporadic glioma has defined molecularly and clinically distinct disease subclasses, based primary on the presence or absence of *IDH1/2* mutation and co-deletion of the p arm of chromosome 1 and the q arm of chromosome 19 (1p/19q codeletion) [1, 2]. Approximately 5% of gliomas occur in individuals with documented family history, defined as having two or more family members with a glioma, These rare cases are likely responsible for a small portion of the samples used by large multi-‘omic’ studies to formally delineate the glioma subclasses designated by an *IDH1/2* mutation and 1p/19q codeletion.

Two recent manuscripts have evaluated a familial glioma case series in light of genomically defined disease subclasses. Our group [3] recently conducted an immunohistochemical and sequencing-based molecular stratification on 163 tumor specimens from individuals with familial glioma, finding a subclass breakdown that largely recapitulated that of sporadic glioma. Ruiz and colleagues [4] reported similar results in a partially overlapping sample cohort derived from 75 individuals with familial glioma, including paired affected individuals within 10 families. This latter analysis additionally identified concordance of subtypes within families (70%), supporting the notion of underlying genetic predisposition for specific disease subtypes. The frequency of associated germline single nucleotide polymorphisms (SNPs) previously identified in glioma genome-wide association studies [5] was similar between familial and sporadic glioma cases [3]. Together, these studies demonstrate that the somatic characteristics of familial glioma are fundamentally quite similar to those of gliomas arising in the general population.

**Figure 1:**
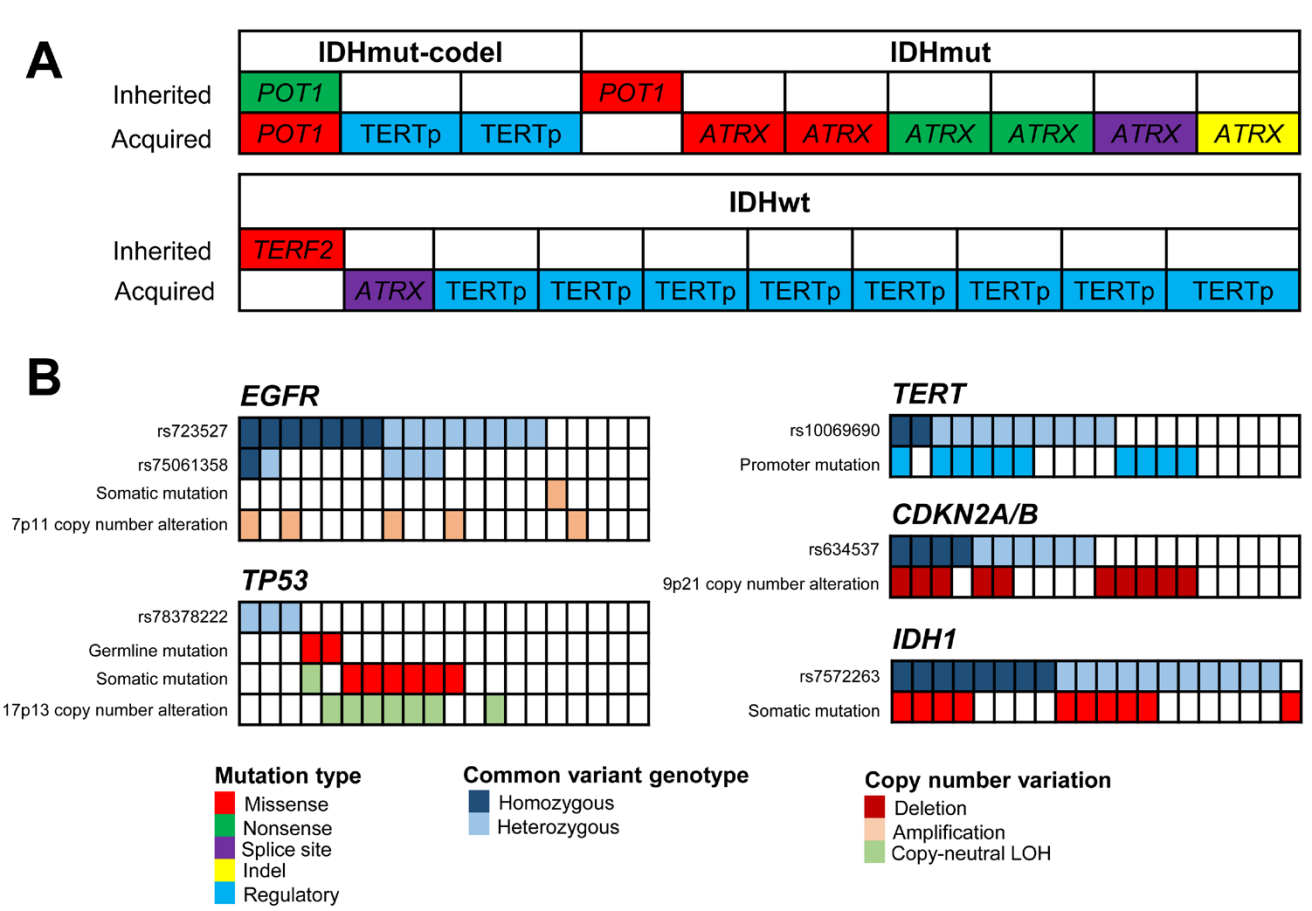
**A.** Mutations in telomere regulatory genes in familial glioma cases, adapted from Jacobs, et al. **B**. Germline risk variations and somatic alterations in *EGFR*, *TP53*, *TERT*, *CDKN2A/B*, and *IDH1* in familial glioma case, adapted from Jacobs, et al.

In our recent study, we also conducted whole exome sequencing on 20 of 163 cases for which patient-matched blood was also available, enabling the identification of both inherited and acquired mutations. Intriguingly, inherited and/or acquired mutations in genes involved in telomere maintenance—*ATRX*, *POT1*, and *TERT—*were identified in all 20 sequenced cases (Figure 1A). The importance of telomere length and function in glioma susceptibility and pathogenesis has been previously described, with both rare and common germline variation in these genes increasing susceptibility for glioma [5, 6]. In addition to multiple hallmark features resulting in altered pathways of telomere maintenance, glioma cells are known to have elongated telomeres as compared to other cancers [7]. In this context, our results emphatically confirm the importance of pathological telomere maintenance in the pathogenesis of adult glioma.

Multiple genes are both frequently altered at the somatic level and harbor germline susceptibility variants in glioma. However, the extent to which germline polymorphisms in glioma influence the acquisition of somatic alterations in adjacent genes remains unclear. Our analysis failed to reveal any correlations between the two, when evaluated tumor by tumor, in either mutation or DNA copy number space (Figure 1B). These findings suggest that inherited risk variants and/or germline mutations do not act as ‘first hits’ with respect to immediately adjacent genes, but rather influence disease pathogenesis through longer range interactions involving more distant genomic sites.

In summary, our results demonstrate the power of integrated analyses involving both germline and somatic genomic profiling in the elucidation of causal molecular pathways of both familial and sporadic glioma. While most glioma genetic susceptibility research to date has been conducted within histologically defined diagnostic categories (e.g. glioblastoma, or lower grade gliomas) [5], recent research has demonstrated associations between specific germline risk variants and molecularly designated disease entities [8]. These insights should lead to a more thorough understanding of how specific genetic risk factors direct pathogenic cascades in the development of molecularly specified glioma variants. We are now continuing this line of investigation in a large set of molecularly classified sporadic gliomas, correlating germline susceptibility variants with patterns of acquired somatic alterations, while also recruiting families with multiple gliomas for parallel analyses. These efforts represent a crucial step in the systematic examination of genetic etiological mechanisms for malignant glioma.
